# Supplementary material for: Impact of Discontinuing Levofloxacin Prophylaxis on Bloodstream Infections in Neutropenic Hematopoietic Stem Cell Transplantation Patients
Source: Antibiotics (Basel). 2022 Sep 19;11(9):1269. doi: 10.3390/antibiotics11091269 (PMC9495722; doi:10.3390/antibiotics11091269)
Supplement: Supplementary file 1 [file antibiotics-11-01269-s001.zip › antibiotics-1869760-supplementary.pdf]

Table S1. Evaluation of risk factors for bloodstream infections (BSI) in neutropenic patients undergoing hematopoietic stem cell transplantation (HSCT) in bivariate and multivariate logistic regression models.

Regression models	Bivariate regression		Multivariate regression [a]	
	Odds ratio (95% confidence interval)	*p*-value	Adjusted odds ratio (95% confidence interval)	*p*-value
Levofloxacin prophylaxis				
Period with prophylaxis (2016 – 2018)	Ref.		Ref.	
Period without prophylaxis (2019)	3.31 (1.84 – 5.94)	<0.001	3.76 (2.00 – 7.06)	<0.001
Gender				
Female	Ref.		Ref.	
Male	1.94 (1.07 – 3.50)	0.03	2.14 (1.13 – 4.04)	0.02
Age	0.99 (0.97 – 1.01)	0.14		
HSCT modality				
Autologous	Ref.			
Allogeneic	0.91 (0.45 – 1.83)	0.79		
Underlying disease				
Multiple myeloma	Ref.		Ref.	
Lymphoma	2.23 (1.14 – 4.39)	0.02	2.21 (1.06 – 4.60)	0.03
Leukemia	1.28 (0.48 – 3.39)	0.62	0.74 (0.24 – 2.32)	0.61
Others	0.48 (0.19 – 1.21)	0.12	0.40 (0.15 – 1.08)	0.07
Duration of neutropenia (days)	1.05 (1.01 – 1.10)	0.03	1.05 (0.99 – 1.11)	0.06

[a] Variables that showed a *p* value <0.1 on the bivariate logistic regression were selected to be included in the multivariate regression model.

Table S2. Evaluation of risk factors for death during hospitalization in neutropenic patients undergoing hematopoietic stem cell transplantation (HSCT) in bivariate and multivariate logistic regression models.

Regression models	Bivariate regression		Multivariate regression [a]	
	Odds ratio (95% confidence interval)	*p*-value	Adjusted odds ratio (95% confidence interval)	*p*-value
Levofloxacin prophylaxis				
Period with prophylaxis (2016 – 2018)	Ref.		Ref.	
Period without prophylaxis (2019)	0.52 (0.15 – 1.87)	0.32	0.23 (0.06 – 0.95)	0.04
Gender				
Female	Ref.			
Male	1.61 (0.58 – 4.47)	0.36		
Age	0.97 (0.94 – 1.01)	0.10		
HSCT modality				
Autologous	Ref.		Ref.	
Allogeneic	6.05 (2.21 – 16.58)	<0.01	5.40 (1.28 – 22.83)	0.02
Underlying disease				
Multiple myeloma	Ref.		Ref.	
Lymphoma	2.44 (0.44 – 13.65)	0.31	0.80 (0.12 – 5.27)	0.81
Leukemia	9.05 (1.67 – 49.08)	0.01	1.14 (0.12 – 11.00)	0.91
Others	4.32 (0.85 – 21.98)	0.08	3.26 (0.52 – 20.55)	0.21
Duration of neutropenia (days)	1.09 (1.03 – 1.15)	0.001	1.04 (0.97 – 1.12)	0.24
Bloodstream infection	5.34 (1.97 – 14.50)	0.001	11.72 (3.16 – 43.43)	<0.001

[a] Variables that showed a p value <0.1 on the bivariate logistic regression were selected to be included in the multivariate regression model and levofloxacin prophylaxis was forced in the multivariate model since it was the main independent variable of interest.

Table S3. Logistic regression analyses evaluating the association between the use of quinolone prophylaxis and 30-day mortality of bloodstream infections (BSI) in neutropenic patients undergoing hematopoietic stem cell transplantation (HSCT) after adjustment for each possible confounding factors in separate.

Regression models	Odds ratio (95% confidence interval)	*p*-value
**Model 1: Age**		
Levofloxacin prophylaxis		
Period with prophylaxis (2016 – 2018)	Ref.	
Period without prophylaxis (2019)	0.10 (0.01 – 0.85)	0.04
Age	0.97 (0.92 – 1.02)	0.23
**Model 2: Type of BSI**		
Levofloxacin prophylaxis		
Period with prophylaxis (2016 – 2018)	Ref.	
Period without prophylaxis (2019)	0.10 (0.01 – 0.83)	0.03
Type of BSI		
Related to central venous catheter	Ref.	
Related to mucosal barrier injury	1.04 (0.23 – 4.74)	0.96
**Model 3: HSCT modality**		
Levofloxacin prophylaxis		
Period with prophylaxis (2016 – 2018)	Ref.	
Period without prophylaxis (2019)	0.11 (0.01 – 0.95)	0.04
HSCT modality		
Autologous	Ref.	
Allogeneic	3.45 (0.69 – 17.36)	0.13
**Model 4: Underlying diseases**		
Levofloxacin prophylaxis		
Period with prophylaxis (2016 – 2018)	Ref.	
Period without prophylaxis (2019)	0.06 (0.01 – 0.58)	0.02
Underlying disease		
Other diseases (except lymphoma)	Ref.	
Lymphoma	0.26 (0.05 – 1.32)	0.10
**Model 5: 3rd generation cephalosporin resistance**		
Levofloxacin prophylaxis		
Period with prophylaxis (2016 – 2018)	Ref.	
Period without prophylaxis (2019)	0.10 (0.01 – 0.93)	0.04
3rd generation cephalosporin resistance		
No resistance to 3rd generation cephalosporin	Ref.	
Resistance to 3rd generation cephalosporin	1.29 (0.27 – 6.12)	0.75
**Model 6: Carbapenem resistance**		
Levofloxacin prophylaxis		
Period with prophylaxis (2016 – 2018)	Ref.	
Period without prophylaxis (2019)	0.11 (0.01 – 0.99)	0.048
Carbapenem resistance		
No resistance to carbapenem	Ref.	
Resistance to carbapenem	4.78 (0.89 – 25.48)	0.068

Table S4. Evaluation of risk factors for 30-day mortality of bloodstream infections (BSI) in neutropenic patients undergoing hematopoietic stem cell transplantation (HSCT) in bivariate and multivariate logistic regression models.

Regression models	Bivariate regression		Multivariate regression [a]	
	Odds ratio (95% confidence interval)	*p*-value	Adjusted odds ratio (95% confidence interval)	*p*-value
Levofloxacin prophylaxis				
Period with prophylaxis (2016 – 2018)	Ref.		Ref.	
Period without prophylaxis (2019)	0.10 (0.01 – 0.82)	0.03	0.12 (0.01 – 1.08)	0.06
Gender				
Female	0.57 (0.14 – 2.42)	0.45		
Male				
Age	0.97 (0.92 – 1.02)	0.18		
Type of BSI				
Related to central venous catheter	Ref.			
Related to mucosal barrier injury	0.71 (0.17 – 2.96)	0.64		
HSCT modality				
Autologous	Ref.		Ref.	
Allogeneic	4.30 (0.94 – 19.58)	0.06	2.80 (0.51 – 15.21)	0.23
Underlying disease				
Other diseases (except lymphoma)	Ref.			
Lymphoma	0.52 (0.12 – 2.31)	0.39		
3rd generation cephalosporin resistance				
No resistance to 3rd generation cephalosporin	Ref.			
Resistance to 3rd generation cephalosporin	2.34 (0.54 – 10.05)	0.25		
Carbapenem resistance				
No resistance to carbapenem	Ref.		Ref.	
Resistance to carbapenem	6.00 (1.25 – 28.74)	0.03	4.03 (0.72 – 22.59)	0.11

[a] Variables that showed a *p* value <0.1 on the bivariate logistic regression were selected to be included in the multivariate regression model.
